# Differential diagnosis of thyroid nodule capsules using random forest guided selection of image features

**DOI:** 10.1038/s41598-022-25788-w

**Published:** 2022-12-14

**Authors:** Lucian G. Eftimie, Remus R. Glogojeanu, A. Tejaswee, Pavel Gheorghita, Stefan G. Stanciu, Augustin Chirila, George A. Stanciu, Angshuman Paul, Radu Hristu

**Affiliations:** 1 Center for Microscopy-Microanalysis and Information Processing, University Politehnica of Bucharest, 313 Splaiul Independentei, 060042 Bucharest, Romania; 2 Pathology Department, Central University Emergency Military Hospital, 134 Calea Plevnei, 010825 Bucharest, Romania; 3 Department of Special Motricity and Medical Recovery, The National University of Physical Education and Sports, 140 Constantin Noica, 060057 Bucharest, Romania; 4 Department of Computer Science and Engineering, Indian Institute of Technology Jodhpur, Jodhpur, India; 5 Faculty of Energetics, University Politehnica of Bucharest, 313 Splaiul Independentei, 060042 Bucharest, Romania

**Keywords:** Thyroid gland, Thyroid cancer, Imaging techniques

## Abstract

Microscopic evaluation of tissue sections stained with hematoxylin and eosin is the current gold standard for diagnosing thyroid pathology. Digital pathology is gaining momentum providing the pathologist with additional cues to traditional routes when placing a diagnosis, therefore it is extremely important to develop new image analysis methods that can extract image features with diagnostic potential. In this work, we use histogram and texture analysis to extract features from microscopic images acquired on thin thyroid nodule capsules sections and demonstrate how they enable the differential diagnosis of thyroid nodules. Targeted thyroid nodules are benign (i.e., follicular adenoma) and malignant (i.e., papillary thyroid carcinoma and its sub-type arising within a follicular adenoma). Our results show that the considered image features can enable the quantitative characterization of the collagen capsule surrounding thyroid nodules and provide an accurate classification of the latter’s type using random forest.

## Introduction

Thyroid nodules, although very common, have no associated symptoms in most of the cases^1^. They are the clinical manifestations of different pathologies^2^, both benign (e.g., hyperplastic colloid nodules or follicular adenoma) and malignant (e.g., papillary thyroid carcinoma, follicular thyroid carcinoma or medullary thyroid carcinoma). Nodular goiter, the most common thyroid lesion, is the result of impaired synthesis of thyroid hormones, most often caused by dietary iodine deficiency^3^. Impairment of thyroid hormone synthesis leads to a compensatory rise in the thyroid-stimulating hormone, which drives the hypertrophy and hyperplasia of the thyroid follicular cells and ultimately leads to the enlargement of the thyroid gland (nodular goiter). Thyroid follicular adenoma (FA) is a benign neoplasm derived from the follicular epithelium. The follicular adenoma is either a solitary lesion that is demarcated from the adjacent thyroid parenchyma through a well-defined, intact collagenous capsule, but can also appear in adenomatous nodular goiters. The capsule surrounding the FA nodule is continuous and made of fibrous tissue, within which vessels of various sizes can be found. The careful evaluation of the integrity of the capsule is critical in distinguishing follicular adenoma from follicular carcinoma. Excision is recommended when there is suspicion that the nodule is malignant, or when the lesion starts to compress the adjacent anatomical structures.

Even though thyroid carcinomas have a low incidence rate, it is essential to distinguish them from adenomas and nodular goiters^4^, as false negative results have dire consequences in this case. The most prevalent form of thyroid carcinoma is the papillary thyroid carcinoma (PTC) accounting for more than 70% of all thyroid cancer cases^5^. PTC is a malignant epithelial tumor showing evidence of follicular cell differentiation and is characterized by a set of distinctive nuclear features (e.g., Orphan Annie eye nuclei)^6^, while the papillae may or may not be present. The lesions can be solitary or multifocal, some may be encapsulated, while others infiltrate the adjacent parenchyma and have ill-defined margins. Surgical excision is the standard treatment, and the diagnosis is confirmed by the optical investigation of hematoxylin and eosin (H&E) stained thyroid tissue sections. The invasion of the capsule is the hallmark of an aggressive form^7^. A subtype of PTC arising within a FA^8^ is a rare histopathological lesion (less than 1% of all PTC cases) that is characterized by groups of cells expressing focally nuclear features of PTC within an otherwise benign FA nodule. This malignant neoplasm may be misdiagnosed following thyroid ultrasound or ultrasound-guided fine needle aspiration biopsy of the thyroid, with the correct diagnosis being made following the microscopic assessment of H&E-stained tissue sections.

The current body of literature provides scarce information about the thyroid nodule capsule, therefore the identification of new cues with diagnostic potential is of utmost importance. To date, the only significant histopathological feature related to the nodule capsule is that it tends to be thicker for malignant nodules^9^ than for benign ones. We have previously shown that combining second harmonic generation (SHG) microscopy^10,11^ with image analysis can provide complementary information to the current histopathological and cytopathological procedures for thyroid pathology. Moreover, by extracting image features from the SHG image sets we were able to differentiate between the thyroid capsule, PTC and FA nodule capsules. Although SHG microscopy with the associated image analysis is already an established method, exhibiting superb specificity for collagen imaging and assessment, its main drawbacks relate to the high cost of the required instrumentation (i.e., fs pulsed laser for excitation) and to the significant difficulties that even expert pathologist encounter when attempting to interpret the outputs of this imaging methodology not documented in any of the histopathology diagnostic guidelines. Although recent progress in digital staining^12,13^ addresses this latter problem, this domain is still in its infancy, coping with specific bottlenecks such as training data availability. All these drawbacks interfere with the translation of experimental SHG methods to clinical environments, where costs and ease of image interpretation are of great importance.

Qualitative evaluation of H&E-stained tissue sections is often sufficient for routine diagnostic purposes, but the advent of digital image analysis techniques and their evolution over the past years^14,15^ hold great promise for future better and faster hybrid diagnostic methods where the objective quantification of microscopic image features will complement and augment the interpretation of human experts. Such quantitative image analysis methods were already applied on whole slide images (WSIs) as a whole^16^ or only on image tiles^17,18^ cropped from WSIs as well as on images acquired using bright-field microscopes^19^. Recent image analysis frameworks^20,21^ developed to match the specifics of microscopy images are gaining increasing momentum and are likely to play an important role in next-gen histopathology.

In this study, we extract features from image tiles cropped from whole slide scanner-acquired images of thyroid nodule capsules. The targeted thyroid pathologies were follicular thyroid adenomas and papillary thyroid carcinomas (with its subtype arising within a follicular adenoma). The statistical analysis and the tested supervised classification approaches indicate that images acquired on the collagen capsules surrounding benign and malignant thyroid nodules have a great potential for differential diagnosis in thyroid pathology.

## Materials and methods

### Sample preparation and imaging

We prepared the thyroid tissue samples for this study according to standard histology protocols. Briefly, the sample preparation involved cutting thin sections (4–7 μm) from the formalin-fixed, paraffin-embedded tissue blocks and mounting them on glass slides. The final step was staining the tissue sections with H&E. We prepared all samples in a single batch to minimize the effect of sample preparation procedures that might result in stain-uptake and color variations. The involved pathologists selected the patients included in this study: seven patients diagnosed with follicular adenoma (FA), seven diagnosed with papillary thyroid carcinoma (PTC) and five patients diagnosed with papillary thyroid carcinoma arising within a follicular adenoma (PTCFA). The ethics committee from the Carol Davila University Central Emergency Military Hospital, Bucharest, Romania approved the use for scientific research purposes of patient samples (protocol number 380/09.06.2020). Written informed consent was obtained from all the patients. All methods were performed according to relevant guidelines and regulations and in accordance with the Declaration of Helsinki.

**Figure 1:**
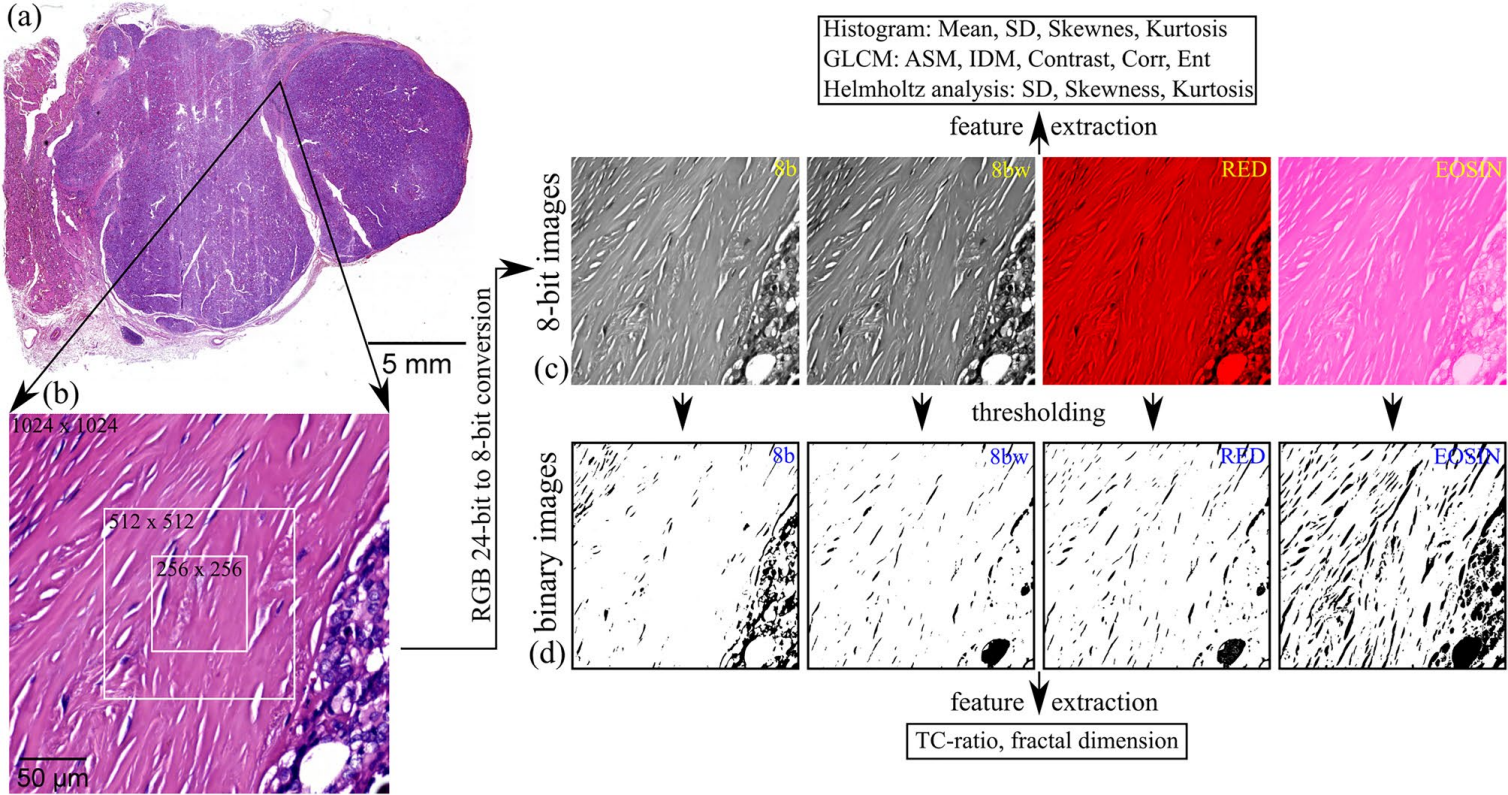
The feature extraction workflow: (**a**) whole slide image acquisition; (**b**) cropping of image tiles with different sizes (i.e., 1024 × 1024, 512 × 512, 256 × 256) within nodule capsules areas; (**c**) RGB to 8-bit conversion using four strategies (i.e., 8b, 8bw, RED, EOSIN) and subsequent feature extraction using the histogram, GLCM and Helmholtz analysis; (**d**) binarization of 8-bit images and subsequent feature extraction (i.e., TC-ratio, fractal dimension).

We imaged the slides with a bright-field Aperio LV1 IVD Whole Slide Scanner (Leica Biosystems) using a 20 × objective lens. We randomly selected regions of interest (ROIs) on collagen capsules surrounding thyroid nodules from the WSIs and cropped image tiles with sizes of 1024 × 1024, 512 × 512 and 256 × 256 pixels from the WSIs, with each consecutive smaller image cropped in the center of the previous (Fig. 1). A total of 736 ROIs were considered: 280, 240 and 216 for each of the involved pathologies, FA, PTC and PTCFA, respectively. Finally, we performed feature extraction using all the obtained image tiles.

### Extraction of image features

To characterize bright-field image tiles cropped from the WSIs we used the same image features as those previously extracted from SHG microscopy images^11^. All these parameters require 8-bit images as input, while the RGB color images obtained using the bright-field whole slide scanner are 24-bit images composed of 3 individual 8-bit channels: red (R), green (G) and blue (B). To extract the features from the bright-field WSIs, we converted the RGB images to 8-bit images by using four different strategies implemented in custom ImageJ^22^ macros.

1. We used the intensity image, and we call this procedure of unweighted RGB to 8-bit conversion as *8b* throughout the manuscript.
2. The second procedure involved a weighted conversion. The default weighting factors used in ImageJ for weighted RGB conversions are 0.299, 0.587, 0.114 for each of the 3 color channels R, G and B respectively. These values are based on human perception. This procedure is called *8bw* in the following.
3. We also selected the red channel as the 8-bit image which is also a weighted conversion, where the weights for the G and B channels are zero. The choice for the red channel was motivated by the fact that eosin stains connective tissue, including collagen, in different shades of red and pink. Images obtained using this procedure are called *RED* in the following.
4. The final method of obtaining the 8-bit image was color deconvolution which we performed by using an ImageJ plugin^23^. Color deconvolution is a method used to transform color bright-field microscopy images acquired on biological samples involving multiple stains into images representing single stain concentrations^24^. Color deconvolution thus allows unmixing 3-channel RGB bright-field images into three individual channels, each of these representing the absorbance of the individual dyes used to stain the sample. In the case of optical images acquired on H&E-stained tissue, two channels correspond to hematoxylin and eosin, respectively, while the third channel does not represent real color information, but the residual of the deconvolution process. It should be empty if the chosen color vectors characterizing hematoxylin and eosin perfectly match the image. From the three output 8-bit channels retrieved from the color deconvolution plugin (i.e., the eosin, hematoxylin, and residual channel) we only used the channel corresponding to the eosin stain which binds among other tissue structures to collagen. Images obtained using this process are called *EOSIN* in the following.

To extract features from the 8-bit images obtained using the above conversion methods, we used different strategies (Fig. 1c) which we describe in the following. A first set of features involved histogram-related parameters (*Mean*, *Standard deviation*, *Skewness* and *Kurtosis*). The second set involved image texture analysis, which we performed using a custom written ImageJ macro to compute the gray-level co-occurrence matrix (GLCM). The macro retrieved five textural parameters previously suggested as relevant for the characterization of collagen in SHG images^11^. The method used to build the GLCM is detailed elsewhere^11,25^, while the five parameters used to characterize the matrix are: *Angular Second Moment* (*ASM*), *Inverse Difference Moment* (*IDM*), *Contrast*, *Correlation* and *Entropy*. The third approach was Helmholtz analysis^26^ performed using an ImageJ plugin which can determine local direction of structures in the images. The plugin returns an image with the direction of structures computed at pixel level. We characterized the Helmholtz orientation image with the same set of first order statistical moments as for the first set of features: *Standard deviation (SD)*, *Skewness* and *Kurtosis*. We didn’t consider the mean values in this case because the average orientation would depend on the orientation of the sample relative to the imaging plane.

The fourth and final set of features involved the binarization of all 8-bit images in ImageJ using the auto histogram-based thresholding procedures (Fig. 1d), with the best result retrieved by the Triangle algorithm. We extracted two additional features from the binary images: the total collagen area ratio (*TC*-*ratio*)^11^ and *Fractal Dimension* (*FD*)^27^. TC-ratio is defined as the ratio between the number of non-zero pixels in the binary image and the total number of pixels in the image, while FD, which is a measure of complexity in the image, was computed using the Fractal Box Count plugin in ImageJ.

### Statistical analysis

For the statistical analysis we used one-way ANOVA followed by Holm multiple comparisons test performed in Prism 9 (GraphPad Software, USA). All statistical tests used a p-value of 0.05 to establish statistical significance. We conduct the feature selection tests by computing the Fisher score^28^.

### Classification

**Figure 2:**
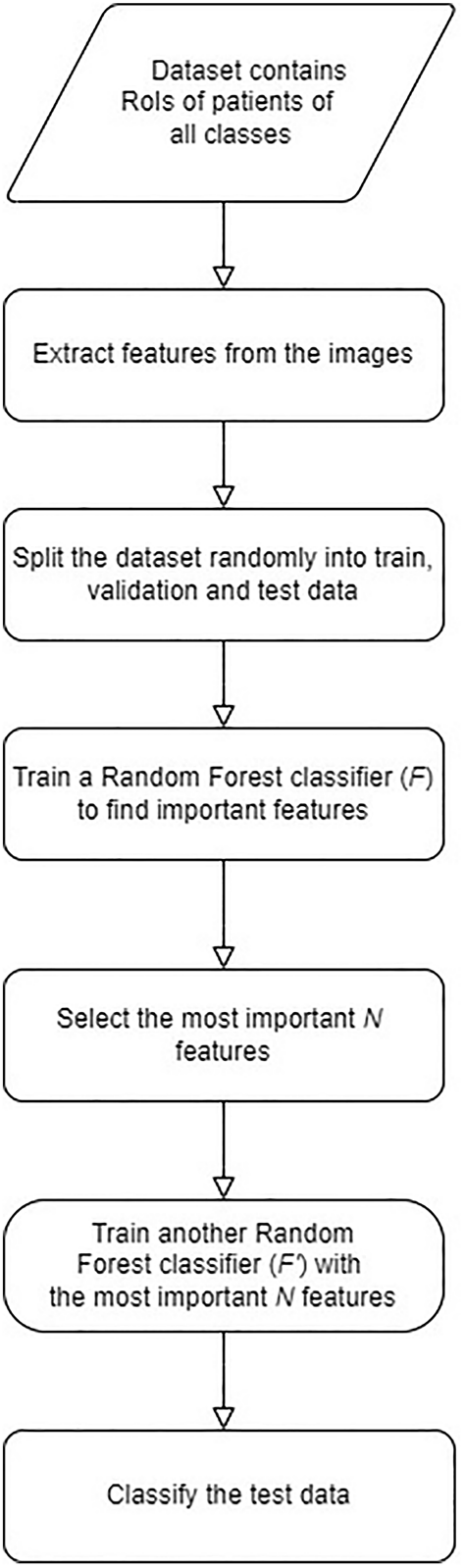
Flowchart showing the experimental steps of the proposed method.

We classify the images into three categories, namely, PTC, FA, and PTCFA using the extracted features. In designing our method, we consider that the extracted features may not all be important in classifying the images and that some of these features may be redundant. Consequently, we performed classification based on the important features. We have thus adopted a two-step approach for classification. First, we find the important features from the set of extracted features. Next, we perform classification using the important features only. Random forest^29^ was selected as the solution that fits best the specifics of both steps described above, considering that we have only several hundred data points for training, which may interfere with the performance of data-hungry approaches like neural networks. In the next we provide additional details on the proposed approach with a flow chart (Fig. 2) presenting the main steps of the experiment.

#### Random forest

A random forest is constructed using several random decision trees grown in parallel. For growing each tree, a random subset of *D* training data is chosen with replacement. In each node of such a decision tree, a random subset of *m* features is initially chosen. Subsequently, a winner feature from this subset splits the node into two children. The winning feature is chosen in a deterministic manner to maximize the homogeneity in each child node. We use Gini impurity^30^ criteria to choose the winner feature and also as a measure of homogeneity in our design. The homogeneity of a node is calculated based on the distribution of data with different class labels in that node. The node splitting is continued until a termination criterion is met. In our design, we chose the number of trees in the forest (*T*) based on the performance of the forest on the validation data. Let us denote this forest as *F*.

#### Finding the important features using random forest

Feature importance can be calculated using random forest through various approaches. We use impurity-based calculation of feature importance using forest *F*. The importance of a feature depends on the total reduction of information due to the splitting of nodes by that feature. If a node in a decision tree contains heterogeneous data (in terms of the class labels of the data), the node has more information compared to a node with homogeneous data. Therefore, upon splitting a heterogeneous node, the feature that creates more homogeneous child nodes causes more reduction of information. A feature that causes more reduction of information across the trees of a random forest is assigned more importance in our computation. This is called Gini importance^30^. Based on the importance values, we take the top *N* most important features for the subsequent classification task.

#### Classification using random forest

Once the *N* important features are identified, we remove all the unimportant features. As a result, we get data points with *N* features and grow another random forest using this dataset with *N* features. Let this forest be denoted as *F'*. Once the forest is constructed, it is used for the classification of the test data. During inference, for the test data, we keep only the *N* features with indices the same as the indices of the important features found in the previous step. Thus, each test data point contains *N* features and is classified into one of the three categories using the trained forest *F'*.

## Results

### Optical imaging and interpretation

**Figure 3:**
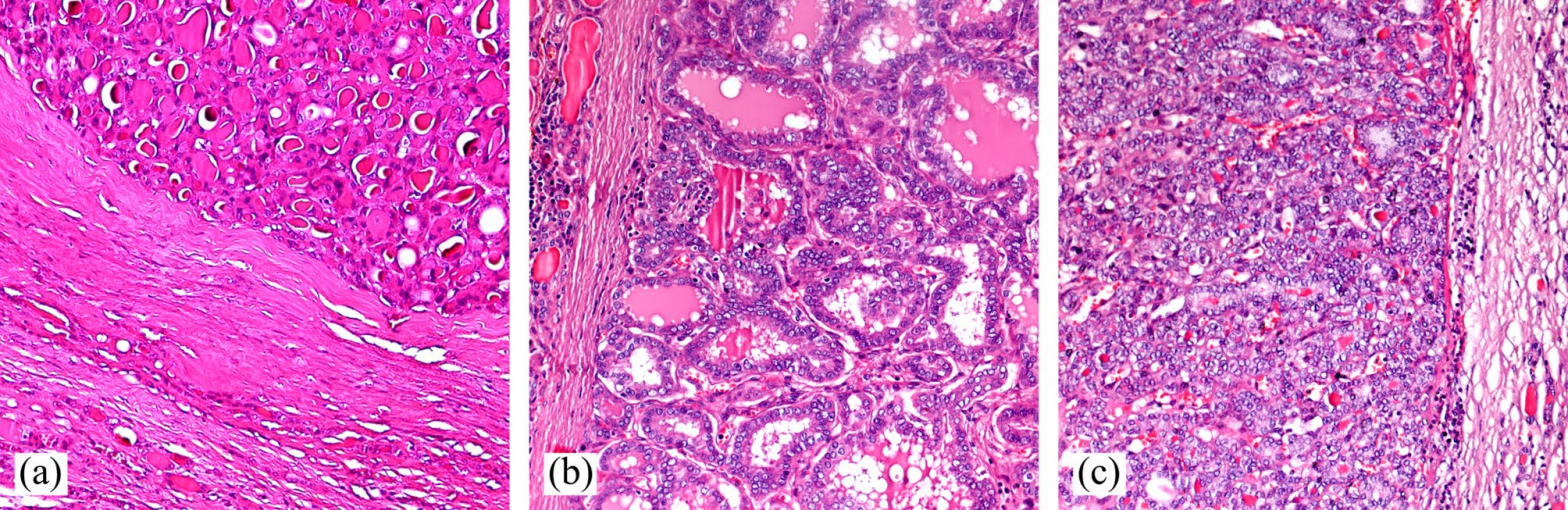
Typical bright-field images acquired on collagen nodule capsules for the cases of (**a**) FA, (**b**) PTC and (**c**) PTCFA.

Figure 3A shows the thickened capsule of an intraparenchymatous nodule from an adenomatous nodular goiter^31^. The nodule exhibits microfollicular (fetal) and trabecular/solid (embryonic) patterns consisting in small thyroid follicles, surrounded by thyroid follicular cells forming a simple cuboidal epithelium. Outside the nodule one can notice the compressed adjacent thyroid parenchyma with discrete diffuse lymphocytic infiltrate. Figure 3b shows another case of thyroid parenchyma compressed by a nodular tumor proliferation, with sclerotic capsule of variable thickness, without capsular invasion, predominantly follicular growth pattern, with cystic dilated neoplastic follicles simulating nodular hyperplasia (macrofollicular variant)^32^. In this case, the characteristic morphological features are the typical papillary nuclear changes of tumor cells: irregular nuclear contours, prominent longitudinal grooves (nuclear pseudoinclusions), and the inner aspect of the compressed chromatin corresponding to an empty appearance of the nucleoplasm^6^. In Fig. 3c we find an encapsulated nodule with thickened capsule. The histopathological examination identified two distinct areas, hence the case was reported as encapsulated variant of PTC arising within a FA^8^, with solid and follicular patterns. Figure 3c exemplifies the area with PTC.

### Statistical analysis of image features

The statistical analysis performed on the features extracted from the image tiles demonstrates that the histogram and the texture analysis using GLCM provide reliable features which can discriminate between the three considered collagen capsules. On the other hand, unsatisfactory results were provided by the TC-ratio and fractal dimension, which were extracted from binary images. Finally, the Helmholtz analysis retrieved features which cannot be used to detect differences between microscopic image tiles acquired on thyroid nodule capsules. Results presented in the following are related to data obtained on 1024 × 1024 pixels image tiles. No significant differences were obtained in the statistical analysis for the 512 × 512 and 256 × 256 pixels images and for simplicity purposes we will refer in the following to the statistical analysis performed on the 1024 × 1024 pixels image tiles.

**Figure 4:**
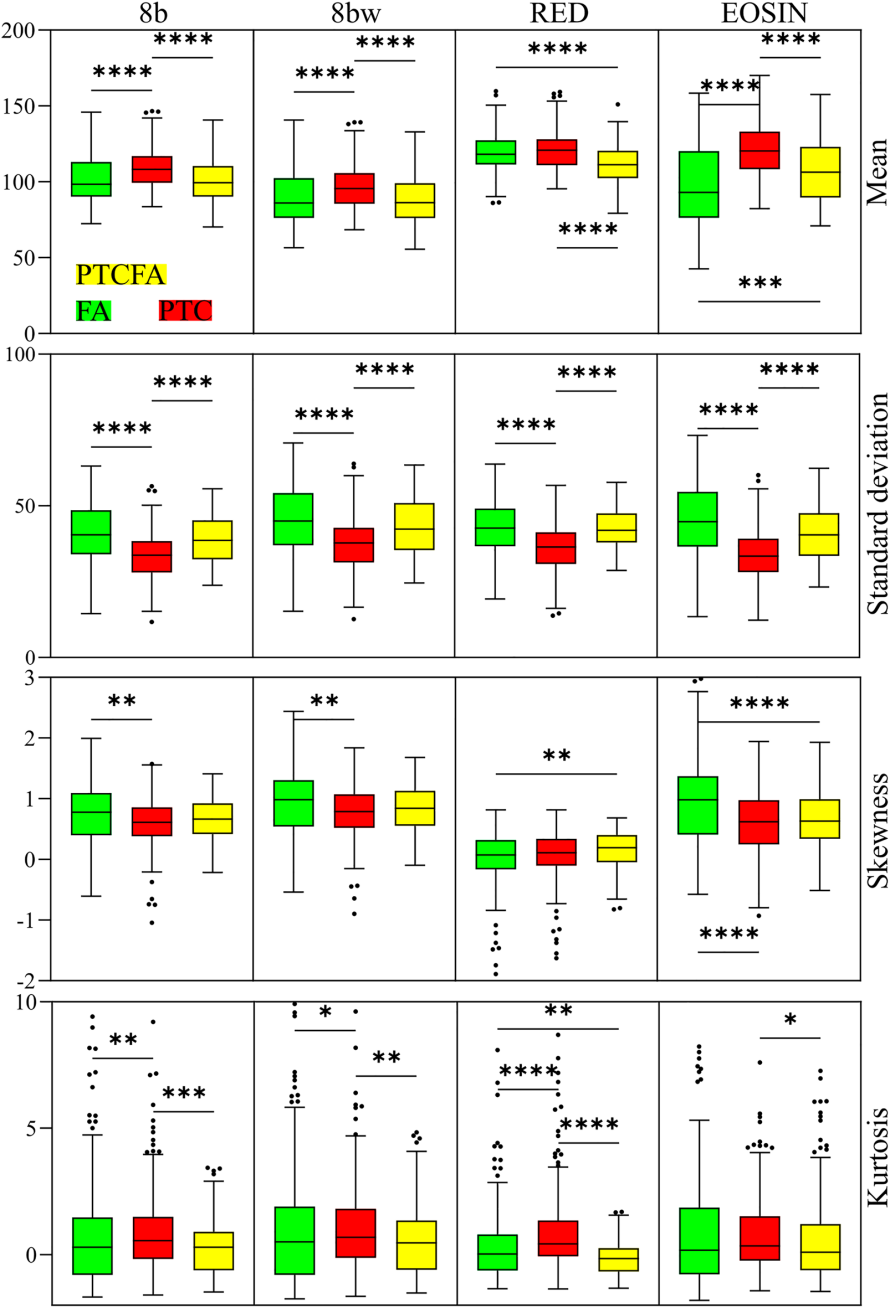
Statistical analysis for the case of histogram parameters extracted from the 1024 × 1024 pixels image tiles. (*p < 0.05, **p < 0.01, ***p < 0.001, ****p < 0.0001).

For all the four considered strategies for RGB to 8-bit image conversion, the highest mean pixel intensities were obtained for image tiles acquired on PTC nodule capsules. Results indicate a statistically significant lower value for the PTCFA capsules, with the lowest mean pixel values obtained for FA nodule capsules. Only for the RED channel, the mean pixel value in images acquired on FA nodules was higher than the value obtained for the PTCFA case. All the differences between mean intensities were statistically significant (Fig. 4). A higher mean pixel value indicates a brighter image. Previously^33^, optical density (OD) which is a measure of the absorbance of light through a sample was used to assess the quality of H&E staining. Because OD is proportional to the stain density, the greater the amount of stain present in the tissue section translates in a greater OD, hence a darker image. Normal OD ranges observed by Chlipala et al.^33^ were higher for hematoxylin than for eosin when using routine 4 μm-thick sections and the standard staining protocol. Thus, one would expect to get a brighter image when eosin-stained tissue components are dominant in an image compared to hematoxylin-stained components. At the same time, brighter images are obtained for cases where tissue gaps are imaged. Because the image tiles were intentionally cropped from collagen areas, they contain little areas with a high nuclear density (i.e., hematoxylin-stained), hence higher mean intensity values might be an indication for a looser collagen nodule capsule for PTC and a more compact one for PTCFA. We excluded the influence of other parameters related to sample preparation^33^ since we prepared the samples in a single batch with the same reagents.

The standard deviation indicates the tendency of the values in a data set to spread around the mean value. Reporting the SD in an image is one way to quantify contrast^34^, with higher SD appearing in higher contrast images. In our experiment, SD provided statistically significant differences when comparing FA and PTC as well as for PTC and PTCFA nodule capsules.

Skewness and kurtosis are measures of histogram shape. The advantage of using distribution shape features stands in the fact that they are intensity independent and are more robust to characterize microscopic images.

**Figure 5:**
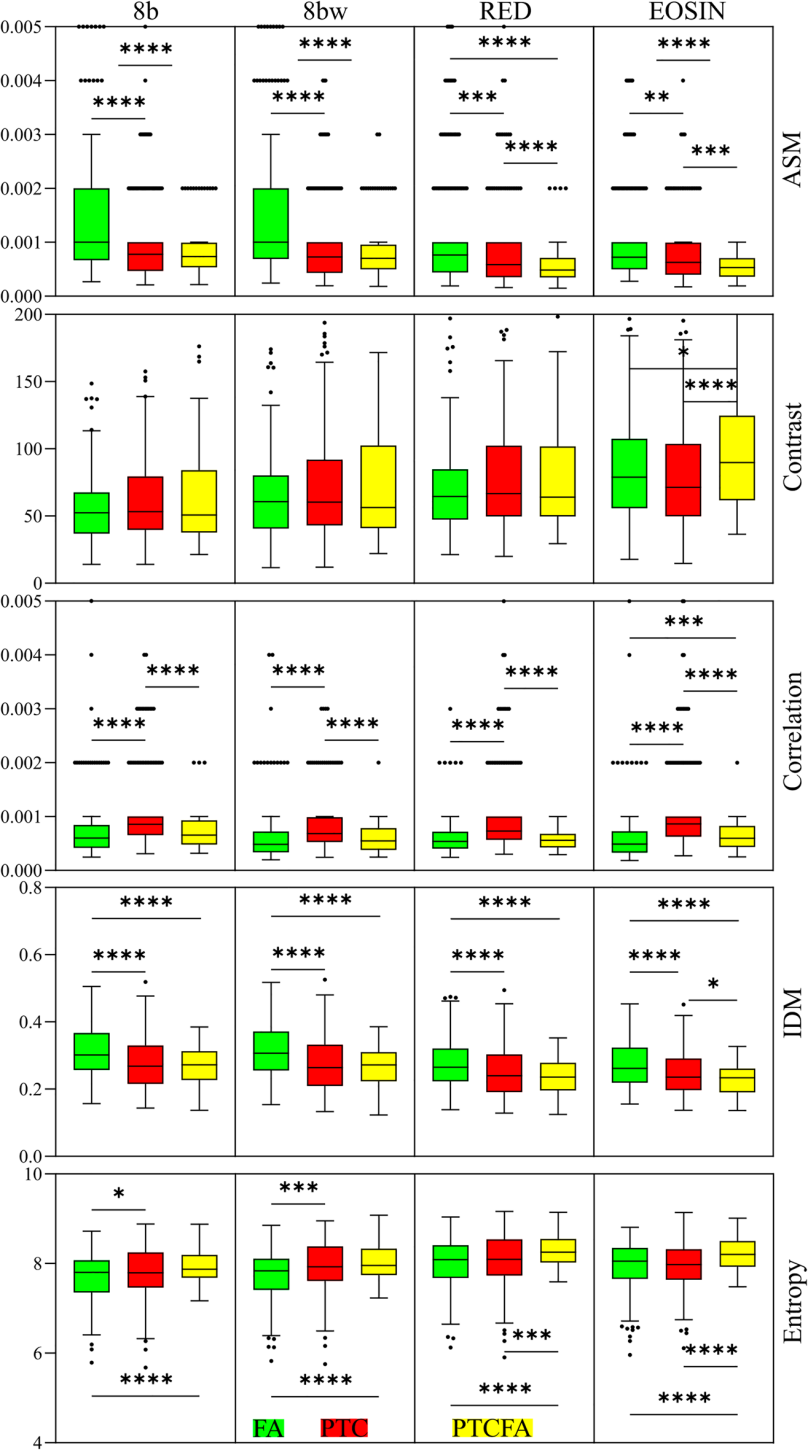
Statistical analysis for the case of GLCM parameters extracted from the 1024 × 1024 pixels image tiles. (*p < 0.05, **p < 0.01, ***p < 0.001, ****p < 0.0001).

While the histogram features are directly related to the pixel intensity distributions, texture analysis features (i.e., features extracted from the GLCM) depend on the spatial relation between pixel intensities. We have computed the GLCM and for each of the images we extracted five features. As expected^35^, *IDM* and *Contrast*, on one hand and *ASM* and *Entropy*, on the other hand are inversely correlated (Fig. 5). Nonetheless, *IDM* seems to perform better than *Contrast*, while *ASM* is better than *Entropy*. In terms of statistical analysis, *IDM* is providing the best results, retrieving statistically relevant differences between any two of the collagen capsule types involved in our experiment (Fig. 5).

The correlation between pixels can provide information about the possible linear relationship between two neighboring pixels within the images. A low *Correlation*, like the one obtained in our case, means a low predictability of pixel relationships. On the other hand, because correlation involves a different calculation method from that of other texture measures^35^, it is independent of these and can retrieve different information.

**Figure 6:**
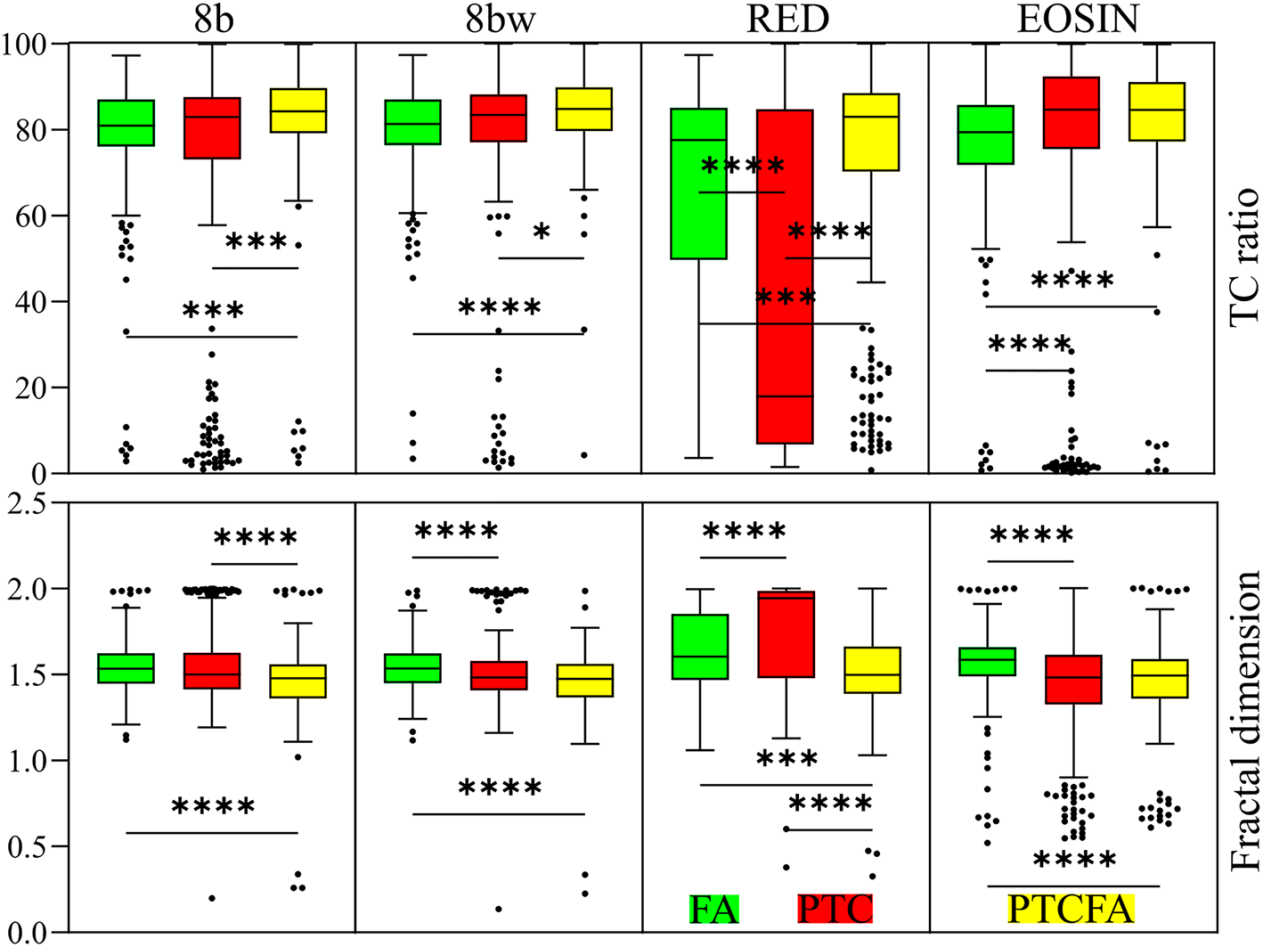
Statistical analysis for the case of binary image parameters extracted from the 1024 × 1024 pixels image tiles. (*p < 0.05, ***p < 0.001, ****p < 0.0001).

Fractal dimension is another feature which can characterize image texture using a binarized version of an image. The RGB image tiles converted to 8-bit images by using the four strategies were further binarized to compute the FD. In three out of the four 8-bit image types used in this experiment, the FD can provide statistically different results when comparing FA vs. PTCFA and PTC vs. PTCFA (Fig. 6), with the highest FD value being obtained for the FA nodule capsules while the lowest for the PTCFA nodule capsule. With fractal dimension being a measure of image complexity, the higher FD values obtained for FA nodules image tiles are aligned with previous results on SHG images^11^ where a correlation between the high FD and a wavy collagen structure was proposed.

The other feature extracted from binary images is TC-ratio, a feature which was previously used in conjunction with SHG images to characterize collagen content^11^. Even though bright-field microscopy of H&E-stained tissue sections might lack specificity to collagen, our results indicate statistically significant lower values in all four studied 8-bit image types for PTC compared to PTCFA nodule capsules. Nevertheless, this quantitative data should be taken with caution, since binarized bright-field images are not a one-to-one representation of the collagen content. Collagen segmentation is not a straightforward task using bright-field optical microscopy images alone, and might require additional steps for processing and image acquisition^36^. Hence, the name of this feature is not correlated with the physical interpretation, but with the previous use on SHG images^11^.

**Figure 7:**
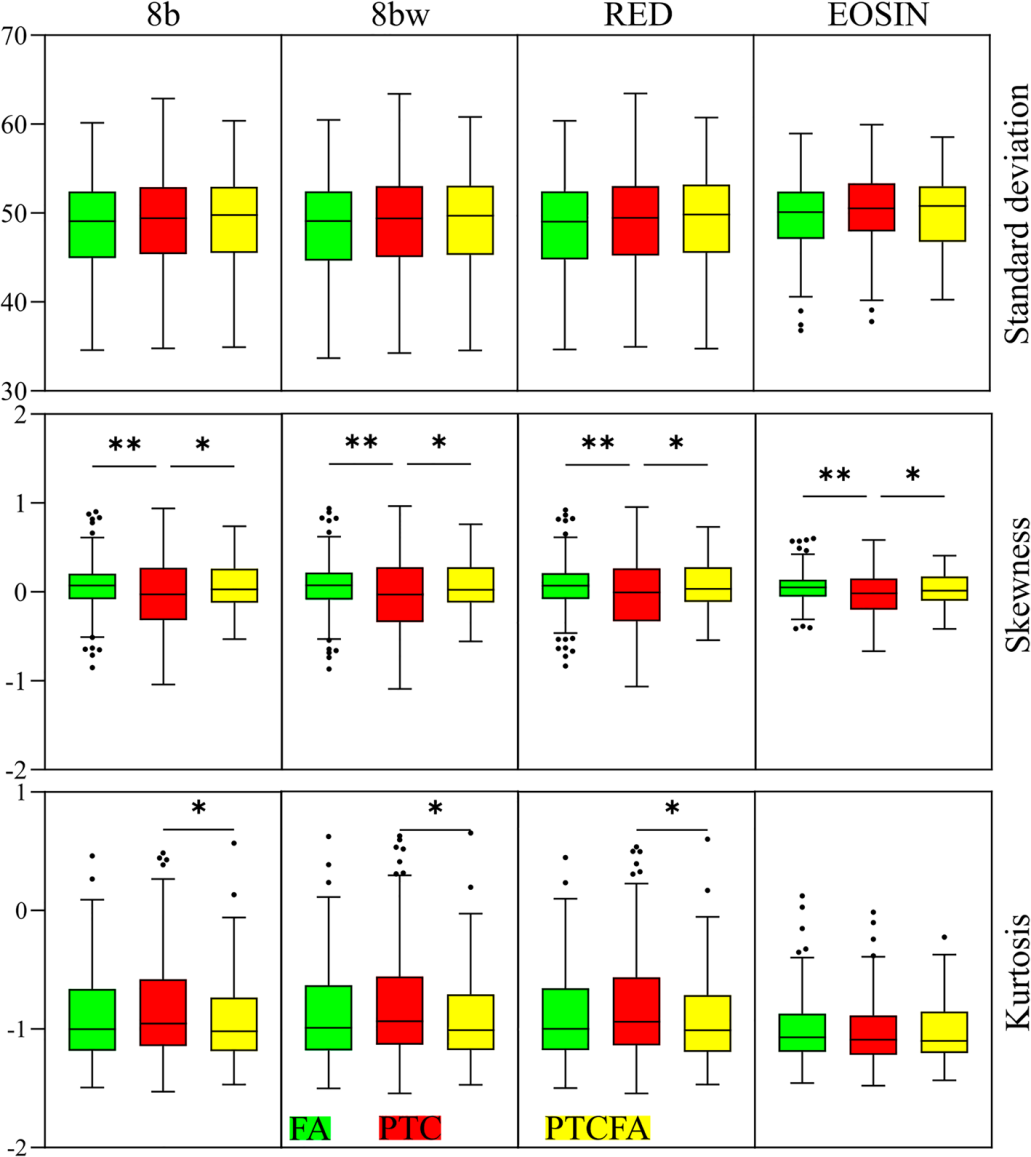
Statistical analysis for the case of Helmholtz analysis parameters extracted from the 1024 × 1024 pixels image tiles. (*p < 0.05, **p < 0.01).

Features extracted using the Helmholtz analysis (Fig. 7) did not demonstrate statistically significant differences between the three considered capsule classes. Helmholtz analysis can be used to estimate the wavelength distribution in an image with periodic features, as well as provide a local estimate for the orientation of the structures within the images. In our case, the poor performance of Helmholtz analysis might be explained on the lack of specificity of optical microscopy images to collagen.

A broader perspective on the image features can be attained by computing the Fisher score, which is one of the most widely used supervised feature selection methods. The Fisher score was previously used for multiclassification^37^ considering the average value of scores for each feature as a threshold for feature selection. For our experiment, we excluded the irrelevant and/or redundant features based on the Fisher score (Table 1), considering only features with Fisher score values above the average Fisher score (0.041).

**Table 1:** Fisher score values for the image features. (*) Fisher scores above the mean Fisher score value (0.041). For comparison, top-10 important features obtained using random forest are underlined.

	256 × 256				512 × 512				1024 × 1024			
	8b	8bw	RED	EOS	8b	8bw	RED	EOS	8b	8bw	RED	EOS
**Histogram**												
Mean	0.05*	0.04*	0.04	0.18*	0.13*	0.12*	0.10*	0.22*	0.08*	0.06*	0.09*	0.18*
SD	0.04	0.03	0.05*	0.07*	0.06*	0.05*	0.07*	0.10*	0.14*	0.12*	0.15*	0.24*
Skewness	0.01	0.01	0.00	0.05*	0.01	0.02	0.00	0.05*	0.02	0.02	0.02	0.07*
Kurtosis	0.03	0.03	0.03	0.02	0.02	0.02	0.04*	0.02	0.02	0.01	0.10*	0.01
**Binary images**												
TC ratio	0.02	0.01	0.06*	0.01	0.04	0.02	0.09*	0.01	0.05*	0.02	0.13*	0.02
FD	0.01	0.01	0.05*	0.01	0.03	0.02	0.08*	0.02	0.04*	0.04	0.09*	0.04
**GLCM**												
ASM	0.03	0.04	0.02	0.04	0.04	0.05*	0.04	0.05*	0.08*	0.09*	0.07*	0.08*
Contrast	0.02	0.02	0.01	0.01	0.01	0.01	0.01	0.01	0.01	0.01	0.01	0.02
Correlation	0.01	0.01	0.03	0.01	0.02	0.01	0.05*	0.01	0.08*	0.06*	0.12*	0.14*
IDM	0.06*	0.07*	0.05*	0.08*	0.04	0.05*	0.04	0.06*	0.08*	0.09*	0.06*	0.11*
Entropy	0.03	0.04*	0.03	0.03	0.02	0.03	0.06	0.03	0.04*	0.06*	0.05*	0.06*
**Helmholtz analysis**												
SD	0.00	0.00	0.00	0.01	0.00	0.00	0.00	0.01	0.00	0.01	0.01	0.01
Skewness	0.02	0.02	0.02	0.02	0.02	0.02	0.02	0.02	0.02	0.03	0.03	0.03
Kurtosis	0.01	0.01	0.01	0.00	0.01	0.01	0.01	0.00	0.01	0.02	0.01	0.00

Similar with the results of the statistical analysis, the features extracted using Helmholtz analysis and *Contrast* in GLCM have Fisher scores under the threshold, hence might be considered unsuitable for the classification. The analysis of the Fisher scores returned the best results in the case of the mean pixel value and the SD computed from the EOSIN image. Other three features extracted from the GLCM (i.e., *ASM*, *Correlation* and *Entropy*) indicate good results, especially for large image tiles, while *IDM* might provide a good classification performance, having a Fisher score above the threshold for almost all the considered situations.

If we consider the influence of the image tile dimension on the feature extraction, the best performance is obtained for 1024 × 1024 pixels image tiles with 30 features above the Fisher score threshold. On the other hand, 17 and 13 features have Fisher scores above the threshold for 512 × 512 and 256 × 256 pixels image tiles, respectively. This result suggests that larger image tiles which, apart from the capsule itself, might also include normal adjacent thyroid tissue and nodular neoplastic cells proliferation are performing better in terms of image feature extraction.

### Classification for differential diagnosis

We used random forest to classify image tiles cropped with different sizes from the WSIs. However, based on the validation performance, we choose the features extracted from 1024 × 1024 images for the classification task. For our experiments, we perform a patient-level splitting of the dataset. We divide the dataset in such a way that data from one patient ID is included only in one of the training, validation, and test sets. This approach prevents leakage of information across splits.

Based on the validation performance, we use a random forest classifier with 450 trees for important feature selection and for classification using only the important features. Starting with the initial forest *F* and the training data, we search for the best features for classification. We thus perform experiments with top *N* features by varying the value of *N*. We perform experiments with the top 20 to top 50 features with an interval of 10, alongside performing experiments with all the features (56 features). Validation performance in these experiments shows the superiority of using the top 30 features for classification. With the set of top 30 important features, we train another forest *F'* using the training data points with these top 30 important features. The results of the proposed method are presented in Table 2 in terms of recall, precision, and F1 score. We have also performed comparisons with several competing methods including KNN classifier^38^, Support vector machine (SVM) (linear and RBF kernels)^39^, and AdaBoost^40^. The performance of the competing methods is evaluated using all the features. Noteworthy, in terms of F1 score on the FA class, the proposed method significantly outperforms all the competing methods except KNN. Although the F1 score of KNN for FA class is comparable to that of the proposed method, the F1 scores for the other two classes using KNN is significantly inferior compared to the proposed method. For the PTC class, the performance of the proposed method is better than all its competitors in terms of F1 score. For PTCFA, the mean F1 score using AdaBoost is better than the proposed method. However, the standard deviation of F1 scores for PTCFA using AdaBoost is significantly higher than that of the proposed method as well. This shows that the F1 scores for PTCFA using the proposed method are more consistent than those of AdaBoost. Additionally, the performance of the proposed method on the other two classes are better than those of AdaBoost. Thus, the superiority of the proposed method over its competitors can be easily noticed in Table 2.

**Table 2:** Comparative performances (mean ± SD) of different classification methods.

Classifiers	F1-score			Recall			Precision		
	FA	PTC	PTCFA	FA	PTC	PTCFA	FA	PTC	PTCFA
KNN	0.60 ± 0.08	0.44 ± 0.10	0.11 ± 0.05	0.69 ± 0.12	0.46 ± 0.11	0.09 ± 0.03	0.55 ± 0.09	0.43 ± 0.15	0.23 ± 0.20
SVM (RBF)	0.38 ± 0.26	0.32 ± 0.28	0.10 ± 0.01	0.43 ± 0.23	0.41 ± 0.31	0.11 ± 0.01	0.37 ± 0.27	0.33 ± 0.22	0.09 ± 0.02
SVM Linear	0.54 ± 0.30	0.48 ± 0.22	0.10 ± 0.09	0.56 ± 0.40	0.62 ± 0.35	0.19 ± 0.27	0.69 ± 0.29	0.37 ± 0.24	0.29 ± 0.41
AdaBoost	0.57 ± 0.16	0.43 ± 0.13	0.36 ± 0.21	0.51 ± 0.19	0.46 ± 0.14	0.44 ± 0.24	0.67 ± 0.09	0.42 ± 0.14	0.35 ± 0.25
Proposed	0.59 ± 0.18	0.51 ± 0.11	0.18 ± 0.06	0.58 ± 0.18	0.59 ± 0.12	0.18 ± 0.05	0.61 ± 0.19	0.48 ± 0.16	0.23 ± 0.15

### On the importance of different features

We also investigate the performance of the random forest in identifying the important features from 1024 × 1024 images. To that end, we use the feature importance obtained from the random forest and sort the features based on their importance (top-10 features are underlined in Table 1). Thus, we find that all the top-10 features obtained from the random forest are identified within the manually selected features based on the Fisher score. This shows the utility of automatically evaluating feature importance using random forest in the context of our problem.

## Discussions

We introduce a method that holds significant potential to enable more accurate differential diagnosis of encapsulated thyroid nodules, by exploring information related to the nodule capsule architecture which has not been used to date in quantitative bright-field microscopy image analysis approaches. Concerning current qualitative histopathology assays, a thyroid nodule examined under a light microscope cannot be diagnosed by mere visualization of the capsule delimiting the tumor nodule from the adjacent thyroid parenchyma.

In this experiment we considered thyroid nodules diagnosed as follicular adenoma, papillary thyroid carcinoma and its subtype arising within a follicular adenoma. We acquired whole slide images from thyroid tissue sections stained with H&E and cropped RGB image tiles with different sizes (i.e., 256 × 256, 512 × 512, and 1024 × 1024 pixels) around the collagenous capsule surrounding thyroid nodules. To enable the feature extraction, we used three strategies to convert the original RGB images to 8-bit images, involving different weighted 8-bit conversion methods, while the fourth method was color deconvolution. The latter allows the extraction of only the eosin-stained tissue areas in an 8-bit image. In this experiment we used the histogram, the gray-level co-occurrence matrix, fractal, and Helmholtz analysis for feature extraction, resulting in a total of 56 image features extracted per image tile.

Using image features whose usefulness for thyroid nodule assessment we previously demonstrated in the context of SHG images^11^, we show that bright-field images acquired on thyroid nodule capsules can be used to differentiate between benign and encapsulated malignant thyroid nodules. The results indicate that although our focus of attention was placed on the collagen capsule surrounding thyroid nodules, wider image tiles which include capsule surroundings provide better results. Moreover, using both the Fisher score and random forest to evaluate the features, those extracted from the RED and EOSIN images (Table 1) prove more suitable for a classification task.

The results obtained in the current experiment indicate that the collagen capsule surrounding thyroid nodules holds features which can be explored by optical microscopy with great promise for differential diagnosis of FA and PTC. On the other hand, we obtained unsatisfactory results for the case of PTCFA. PTCFA nodules exhibit histological features which are consistent with both FA (e.g., thyroid follicles) and PTC (e.g., irregular follicular architecture, tumor cells with enlarged nuclei, chromatin clearing, irregular nuclear membrane). Fine-needle aspiration biopsy of PTCFA nodules will detect cells with features of both PTC and FA and may not fully meet the criteria for a cytological diagnosis of either entity^8^. Because we performed a random cropping of image tiles around the nodule capsules, the result for PTCFA was a heterogeneous set of images with features resembling both FA and PTC.

We envision that this approach can be extended to other histopathological types of encapsulated thyroid nodules (e.g., non-invasive follicular thyroid neoplasm with papillary-like nuclear features, follicular thyroid carcinoma, medullary thyroid carcinoma or anaplastic carcinoma) with additional feature extraction and classification strategies.

The feature set extracted from the image tiles can be extended using one of the following strategies:

(i) Different weighting factors for RGB to 8-bit conversion can be tested to improve the results, even considering only the green or the blue color channels.
(ii) The color deconvolution procedure can be improved by determining the staining vectors in one’s own lab from single-dye-stained sections. In the current experiment we used background-corrected, bright-field images and selected the pre-computed vectors for the H&E staining method available with the plugin used for color deconvolution^23^.
(iii) Collagen segmentation from the microscopic image tiles for the TC-ratio and fractal dimension feature extraction can be performed using other methods such as: superpixel approaches^41^ or machine learning tools for pixel classification^42^.
(iv) Other microscopy approaches more specific to collagen than bright-field microscopy (e.g., dual-mode emission and transmission (DUET)^36^, polychromatic polarization microscopy (PPM)^43^) can be bundled with image feature extraction for thyroid nodule capsule classification. DUET was proposed as an alternative to the traditional approaches of using trichrome stains for collagen in tissue samples. It does not require special stains or advanced optical methods and can generate virtual histochemical images that resemble trichrome-stained slides. On the other hand, PPM was used to visualize collagen with an optically generated color representation of fiber orientation and alignment under a regular microscope with minor modifications. While PPM does not require stained slides, it is still compatible with H&E-stained tissue sections, making the integration in routine histology protocols straightforward.

The second set of strategies would involve using deep learning approaches for classification of acquired image tiles. In this regard, convolutional neural network (CNN) models can automatically extract image features preserving the spatial context. Classification is subsequently performed based on such features. To deal with the relative scarcity of training images, one can explore low-shot image classification approaches using CNNs.

Combining bright-field microscopy with image feature extraction can be thus regarded as an additional technique with applicability in pathology which could help avoid under- or over-diagnosis of lesions, whose correct diagnosis is essential for proper and effective treatment. This approach would be a workaround to the excess use of relatively expensive complementary diagnostic techniques (e.g., molecular biology techniques, genetic tests, immunohistochemistry, electron microscopy).

## Conclusions

In this experiment we address the quantitative analysis of bright-field images acquired on thyroid nodules diagnosed as follicular adenoma, papillary thyroid carcinoma and its subtype arising within a follicular adenoma. Image features were extracted from the histogram, texture analysis with the gray-level co-occurrence matrix, Helmholtz, and fractal analysis. Our results suggest that some thyroid encapsulated nodules can be differentiated by studying their capsules, without the need for tumor cell morphology, an indispensable element in the development of histopathological diagnosis of certain tumors. Overall, the present study demonstrates the potential of thyroid nodule capsules as a diagnostic feature that provides complementary information to the current histopathology procedures. We argue that this additional level of information holds important potential to enable better and faster methods for the precise diagnosis of thyroid nodules.

## Data Availability

The datasets generated during and/or analyzed during the current study are available from the corresponding author on reasonable request.
